# Apparent diameter and cell density of yeast strains with different ploidy

**DOI:** 10.1038/s41598-023-28800-z

**Published:** 2023-01-27

**Authors:** Nobuo Fukuda

**Affiliations:** Biomedical Research Institute, National Institute of Advanced Industrial Science and Technology (AIST), 1-8-31 Midorigaoka, Ikeda, Osaka 563-8577 Japan

**Keywords:** Biological techniques, Biotechnology, Microbiology

## Abstract

Optical density at 600 nm (OD_600_) measurements are routinely and quickly taken to estimate cell density in cultivation and to track cell growth. The yeast *Saccharomyces cerevisiae* is one of the microorganisms most used in industry, and the OD_600_ values are frequently adopted as the indicator of yeast cell density, according to the Beer–Lambert law. Because the OD_600_ value is based on turbidity measurement, the Beer–Lambert law can be applied only for microbial cultivation with low cell densities. The proportionality constants strongly depend on several parameters such as cell size. Typically, yeast strains are categorized into haploids and diploids. It is well known that cell size of diploid yeasts is larger than haploid cells. Additionally, polyploid (especially triploid and tetraploid) yeast cells are also employed in several human-activities such as bread-making and lager-brewing. As a matter of fact, there is almost no attention paid to the difference in the proportionality constants depending on the yeast ploidy. This study presents information for cell size of haploid, diploid, triploid, and tetraploid yeasts with isogenic background, and describes their proportionality constants (*k*) corresponding to the molar extinction coefficient (*ε*) in the Beer–Lambert law. Importantly, it was found that the constants are inversely proportional to apparent cell diameters estimated by flow cytometric analysis. Although each cell property highly depends on genetic and environmental factors, a set of results obtained from yeast strains with different ploidy in the current study would serve as a major reference source for researchers and technical experts.

## Introduction

Optical density (OD) measurements of microbial growth are one of the most common techniques used in microbiology^1^. Using ultraviolet–visible (UV–Vis) spectroscopy, the OD measurements are routinely and quickly taken to estimate cell density in cultivation and to track cell growth. The wavelength 600 nm is commonly used due to the optical properties of microbial cultivation media in which cells are grown and to less cell damage for continued experimentation. From the obtained OD value at 600 nm (OD_600_), microbial cell density (*c*) can be calculated with accordance to the Beer–Lambert law (Eq. 1).

1 $${\text{OD}}_{600} = - {\text{log}}\left[ {\frac{I}{{I_{0} }}} \right] = \varepsilon \cdot c \cdot l.$$

In this equation, *I*_*0*_ and *I* mean the incident light intensity (set at 600 nm) and the transmitted light intensity through the sample, respectively. Additionally, *ε* is the attenuation coefficient, and *l* is the pathlength^2^. Because the OD_600_ value is based on turbidity measurement, the Beer–Lambert law can be applied only for microbial culture with low cell densities or adequately diluted samples. The constants (*ε*) strongly depend on several parameters such as cell size^1^.

The yeast *Saccharomyces cerevisiae* is one of the best-understood biological systems and can produce numerous useful compounds. Typically, yeast strains are categorized into haploids and diploids. As compared to those of haploid strains, diploid strains of *S. cerevisiae* are commonly used in brewing industries due to the better fermentation efficiency in terms of vitality and endurance. Haploid cells have a diameter of approximately 4–5 μm^3^, and cell sizes of diploid cells are larger than haploids. Regardless of difference in cell size between haploid and diploid cells, there is almost no emphasis on difference in the attenuation coefficients in the Beer–Lambert law. Moreover, polyploid (especially triploid and tetraploid) yeast cells are also employed in several human-activities such as bread-making and lager-brewing^4–6^. These variations in ploidy have made it difficult to estimate the cell density in various performance evaluation of yeast strains.

2 $$c = \frac{1}{\varepsilon \cdot l} {\mathrm{OD}}_{600}= k {\cdot \mathrm{OD}}_{600}$$

In the current study, the relationship between cell size and proportionality constant *k* (Eq. 2) was investigated using haploid, diploid, triploid, and tetraploid yeast strains with isogenic background. It is impossible to compare the OD_600_ values measured by a different principle. As one of the simplest and easiest means many researchers can use, transmitted light method using UV–Vis spectroscope was utilized for OD_600_ measurement in this study.

3 $$\varepsilon = f(d)$$

Furthermore, information for apparent cell diameter *d* of each yeast strain was acquired from forward scatter (FSC) values in flow cytometry. Using the cell size as a variable, attenuation coefficient *ε* was approximately described (Eq. 3).

## Results

### Investigation of proportionality constant *k*

**Figure 1:**
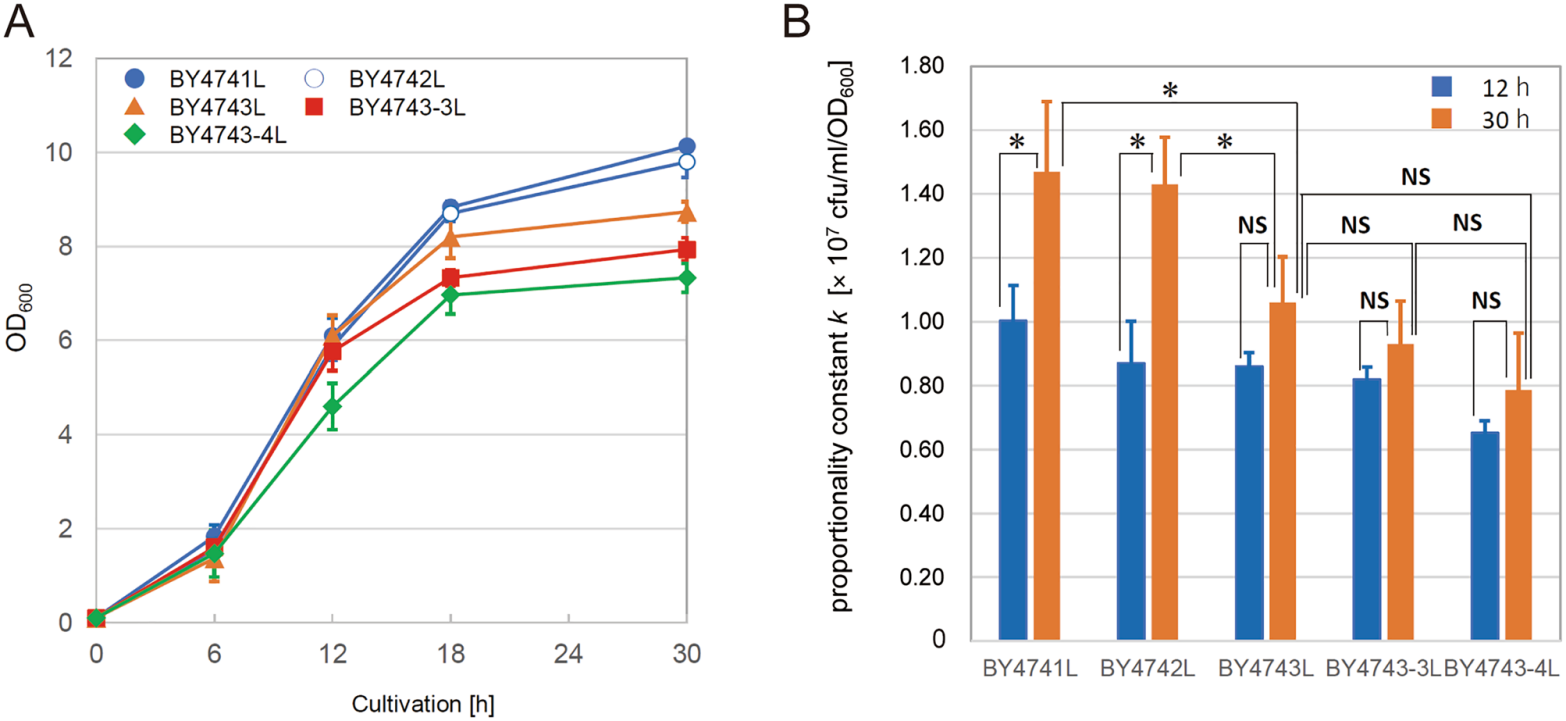
Growth characteristics and proportionality constants *k* values of haploid, diploid, triploid, and tetraploid strains. (**A**) Growth curve of each strain. (**B**) Investigation of proportionality constants *k* values. Yeast cell cultures (12 or 30 h) were diluted (setting OD_600_ at 10^–5^), and then spread on the YPD solid medium. Cell density was estimated by colony counting, and proportionality constants *k* were calculated according to Eq. 2. Values are presented as means ± standard deviations from three independent experiments. Statistically significant differences were determined by Student’s t-test. (NS: not significant, *p < 0.05, **p < 0.01).

Yeast strains BY4741L (haploid)^7^, BY4742L (haploid)^8^, BY4743L (diploid)^8^, BY4743-3L (triploid)^8^, and BY4743-4^8^ (tetraploid) were prepared (Table 1) for comparison of proportionality constant *k* (Eq. 2). The ploidy of each strain was confirmed in the previous studies^7,8^. To maintain linearity between the *c* and OD_600_ values, the measured OD_600_ values must be within the dynamic range of equipment. However, the values frequently reach to 10 at the end of cultivation in the actual OD_600_ measurements^9,10^. In the current study, the OD_600_ values of 10 times diluted samples were measured in time course of cultivation. As shown in Fig. 1A, the OD_600_ value at the end of cultivation decreased as the ploidy increased.

**Table 1:** Yeast strain used in this study.

Name	Ploidy	Description	Reference source
BY4741L	Haploid	*MAT***a** *his3Δ1 ura3Δ0 met15Δ0*	^7^
BY4742L	Haploid	*MAT*α *his3Δ1 ura3Δ0 lys2Δ0*	^8^
BY4743L	Diploid	*MAT***a**/α *his3Δ1/his3Δ1 LEU2/leu2Δ0 LYS2/lys2Δ0 met15Δ0/MET15 ura3Δ0/ura3Δ0*	^8^
BY4743-3L	Triploid	*MAT***a**/**a**/α *his3Δ1/his3Δ1/his3Δ1 LEU2/leu2Δ0/leu2Δ0 LYS2/LYS2/lys2Δ0 met15Δ0/met15Δ0/MET15 ura3Δ0/ura3Δ0/ura3Δ0*	^8^
BY4743-4L	Tetraploid	*MAT***a**/**a**/**a**/α* his3Δ1/his3Δ1/his3Δ1/his3Δ1 LEU2/leu2Δ0/leu2Δ0/leu2Δ0 LYS2/LYS2//LYS2/lys2Δ0 met15Δ0/met15Δ0/met15Δ0/ MET15 ura3Δ0/ura3Δ0/ura3Δ0/ura3Δ0*	^8^
K7A	Diploid	**a**-type of strain derived from Kyokai No. 7	^11^

As described above, the OD_600_ value is not linearly related to cell count except within a limited range^1^. To investigate the effect of growth phase, the proportionality constant *k* of each strain was semi-quantitatively measured over time by colony counting method^11^ (Supporting Information Fig. S1). Based on the results of yeast strains with different ploidy, there were differences in the constant *k* between stationary phase and exponential growth phase. Therefore, the constant *k* of each strain was measured and compared between 12 and 30 h cultivation.

Compared to 30 h cultivation, there was less difference in the constant *k* at 12 h among yeast strains with different ploidy (Fig. 1B). The measured raw OD_600_ values at 12 h ranged from 0.3 to 0.7, whereas that at 30 h ranged from 0.7 to 1.1. A two-fold stepwise dilution series were prepared to search the dynamic range in the current system (Supporting Information Fig. S2A and B). Where OD_600_ > 0.7, the values were underestimated due to excess cell density. On the other hand, the values were overestimated in most cases due to detection noises where OD_600_ < 0.2. Based on the underestimation of OD_600_ value shown in Fig. S2B, the degree of overestimation of *k* at 30 h was roughly calculated (Supporting Information Table S1).

To determine the dynamic range in the OD_600_ measurement in the current system, alternative dilution series were prepared setting the dilution factor at 1.25 (Supporting Information Fig. S2C). Where the raw OD_600_ values ranged from 0.1 to 0.8, coefficients of determination (*R*^2^) were over 0.99. By excluding the fluctuation area (Supporting Information Fig. S2D), the *R*^2^ values were improved to 0.999 where the approximate raw OD_600_ values ranged from 0.3 to 0.8 (Supporting Information Fig. S2E).

Whereas there was statistically significant difference (*p* < 0.05) in the constant *k* at 30 h between haploid and diploid strains, there was no statistically significant difference in that both between diploid and triploid strains, and between triploid and tetraploid strains. Although there was statistically significant difference (*p* < 0.05) in the constant *k* between 12 and 30 h cultivation only in the case of haploid and diploid strains (Fig. 1B), these results might be artifacts derived from the underestimation of the OD_600_ values.

Furthermore, in colony counting method, the number of formed colonies cannot be equal to the number of cells in suspension because the flocculated cells form a single colony. In the current study, the mean value of each measurement was used as the index of population characteristics. Using the OD_600_ and *k* values, cell density (*c*) at 30 h cultivation was calculated according to the Eq. (2) (Supporting Information Fig. S3). The mean value of *c* at the end of cultivation also decreased as the ploidy increased.

### Microscopic observation for cell size

**Figure 2:**
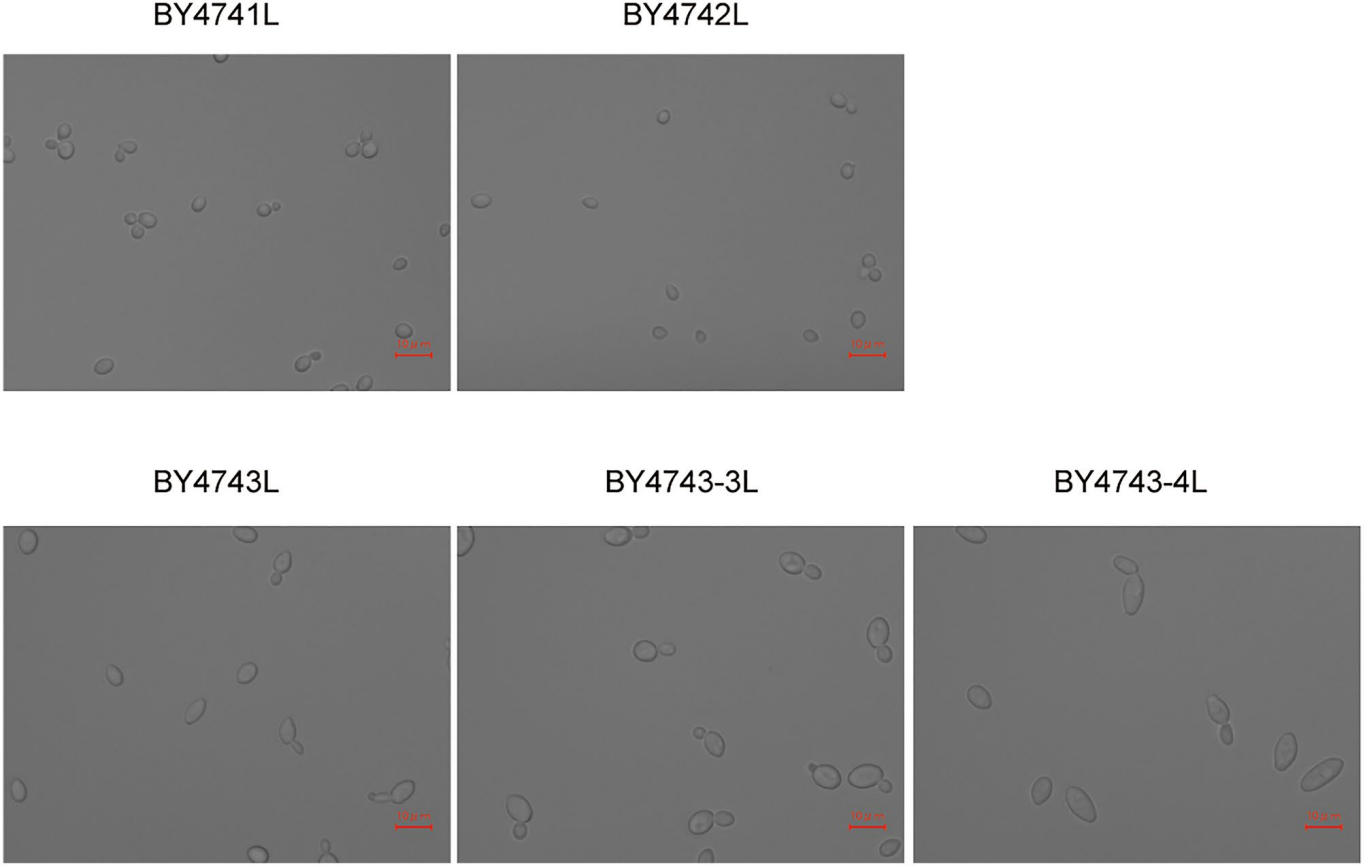
Microscopy images of yeast cells. Scale bar: 10 μm.

In UV–Vis spectroscopy, the output represents total amount of cell populations. Substantially, microscopic observation was carried out to comprehend the approximate size of a single cell of each strain (Fig. 2). In the cases of haploid strains BY4741L and BY4742L, the major axis of most cells was smaller than 5 µm. Unlike other strains, slightly flocculated cells (comprising more than three cells) appeared in observation of haploid cells. The major axis of some diploid cells was larger than 5 µm, and cells with the major axis of approximately 10 µm and > 10 µm appeared in cell population of the triploid and tetraploid strains, respectively. These results indicate that the average cell size of population increased with the ploidy. In all cases, budding cells (comprising two cells) were observed in cell population.

### Comparison of the apparent cell diameter using flow cytometry

**Figure 3:**
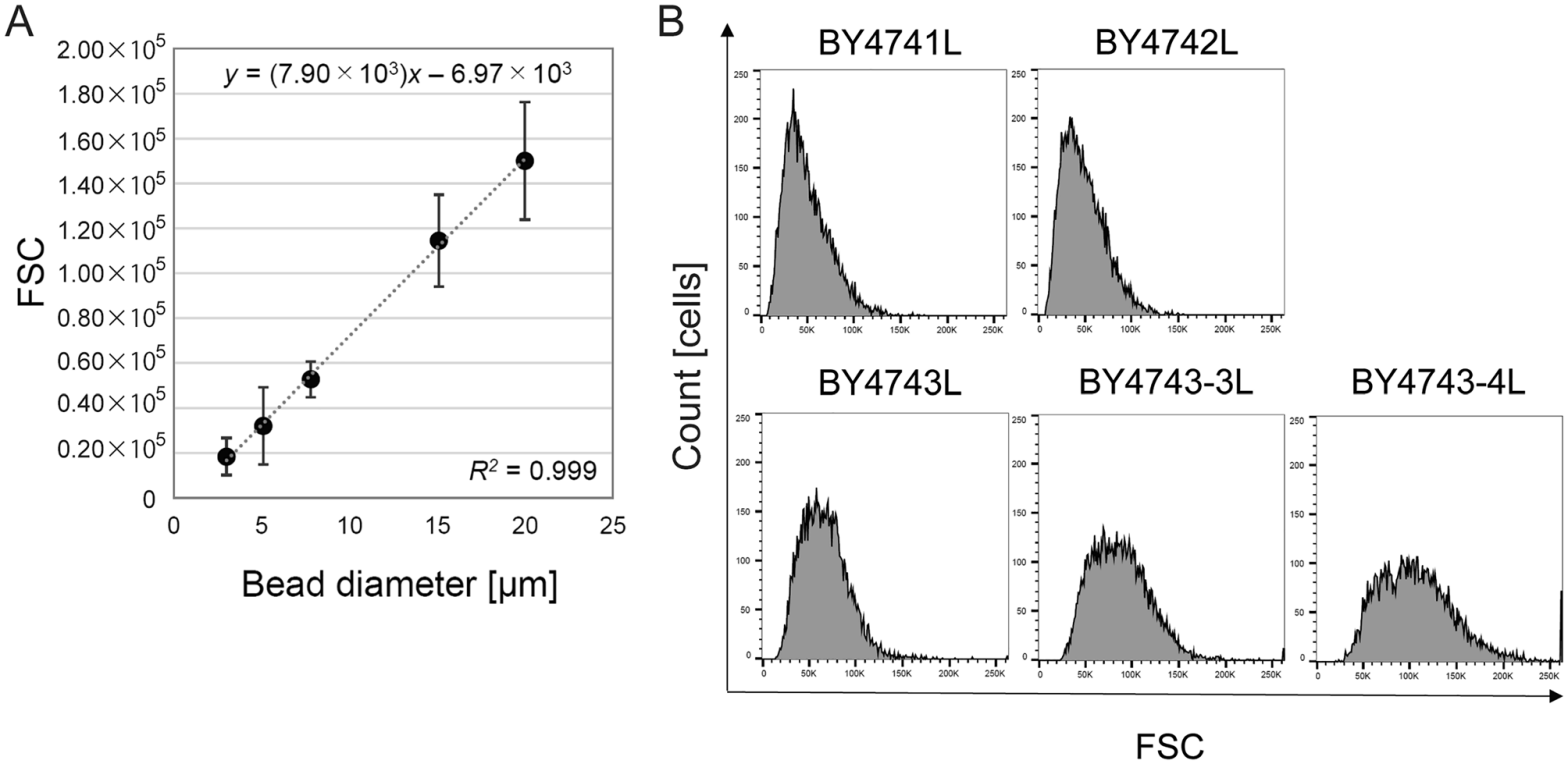
Apparent cell diameter determined using flow cytometry. (**A**) Calibration curve using size reference beads. Values are presented as means ± standard deviations obtained from 10,000 particles. (**B**) FSC histogram of each strain.

In flow cytometry, the measurement of forward scatter (FSC) allows for the discrimination of cells by size, because FSC intensity is proportional to the diameter of the cell^12^. Using size reference beads, standard curve was drawn for the measurements of cell diameter (Fig. 3A). An obvious distribution in the detected FSC values appeared in flow cytometric analyses (Supporting Information Fig. S4), and the mean values of FSC were used as the corresponding values of the diameter of size reference beads.

The FSC histogram of each strain was shown in Fig. 3B. Although each histogram had a tail on the right side (the existence of the flocculated cells in population), the peak position shifted to right with the ploidy. The apparent cell diameters (*d*) were represented by the mean values of populations, and relative diameters (*D*) were calculated as the *d* value of BY4741L as the reference (Table 2). The diameter of the tetraploid strain BY4743-4L was approximately twice that of the haploid strain BY4741L. Because budding and flocculated cells existed in suspension as observed using microscope, the *d* values measured by flow cytometer were larger than the diameters of single cells.

**Table 2:** Relationship between proportionality constant *k* values and apparent cell diameters of yeast cells.

Strain	Proportionality constant *k* [× 10^7^ cells/ml/OD_600_]	Normalized constant (*K*)	Apparent cell diameter *d* [µm]	Relative cell diameter (*D*)	Product (*K* × *D*)
BY4741L	1.47 (± 0.22)	1.00 (± 0.15)	7.3 (± 4.0)	1.00 (± 0.54)	1.00 (± 0.69)
BY4742L	1.43 (± 0.15)	0.97 (± 0.10)	7.1 (± 3.9)	0.98 (± 0.53)	0.95 (± 0.61)
BY4743L	1.06 (± 0.14)	0.72 (± 0.10)	9.4 (± 4.2)	1.29 (± 0.58)	0.93 (± 0.54)
BY4743-3L	0.93 (± 0.13)	0.63 (± 0.09)	12.0 (± 5.0)	1.65 (± 0.68)	1.04 (± 0.58)
BY4743-4L	0.79 (± 0.18)	0.54 (± 0.12)	14.5 (± 6.1)	1.99 (± 0.84)	1.07 (± 0.69)
K7A	0.70 (± 0.11)	0.52 (± 0.07)	12.6 (± 9.4)	1.72 (± 1.29)	0.89 (± 0.80)

The *d* values must have influence on the constants *k*. Like the *D* values, normalized constants (*K*) were also calculated using the constant *k* at 30 h of BY4741L as the reference (Table 2). The values of *K* obviously decreased as the *D* values increased, and then the products of *K* and *D* (*K* × *D*) were compared among yeast strains with different ploidy. It was found that the *K* × *D* values were approximately 1.00 despite the ploidy of yeast strains, which suggests that the constant* k* is inversely proportional to the *d* value although the relationship is just an approximation under certain but general conditions.

4 $${\mathrm{OD}}_{600}= \alpha \cdot d\cdot c\cdot l$$

Approximate formula constructed in the current study is described using apparent cell diameter *d* [cm] (Eq. 4). The estimated value of the constant *α* was 9.38 × 10^–5^ [ml/cells/cm^2^] (Supporting information Fig. S5). To confirm the availability of Eq. (4), it was applied to cultivation of sake yeast strain K7A^13^ at brewing temperature (15 °C). The OD_600_ values were measured in time course of cultivation (Supporting information Fig. S6A), and microscopic observation was carried out at the end of cultivation (Supporting information Fig. S6B). Unlike other strains cultivated at 30 °C, aggregated cells (comprising more than ten cells) obviously appeared in microscopic observation in accordance with flow cytometric analysis (Supporting information Fig. S6C). Although the aggregated cells would form a single colony, the proportionality constant *k* of sake strain K7A (96 h) was quantitatively measured by colony counting method in the same manner as other strains. Furthermore, the apparent cell diameter was determined using flowcytometry. Although the *K* × *D* value of sake strain K7A was slightly lower than other strains (Table 2), Eq. (4) was applicable to the case with different strains and cultivation condition (Supporting information Fig. S6D).

## Discussion

The OD_600_ measurement is widely used to estimate cell density in microbial culture^14^, but it is difficult to compare the OD_600_ values between strains with different cell diameter. Reliable counting the number of cells in a liquid culture has remained a challenge especially in microbiology for decades.

As a premise, the apparent cell diameter in population must be larger than the diameter of a single cell due to budding and yeast cell flocculation^15–17^. In addition, cell size can significantly vary during cell cycle^18^. Yeast cells in suspension would have heterogeneity in diameter unlike uniform sized particles. The degree of heterogeneity in diameter was confirmed by microscopic observation (Fig. 2) and flow cytometric analyses (Fig. 3), and then the apparent cell diameter of each strain was estimated as the average diameter of particles detected by flow cytometer (Table 2).

In principle, the measured OD_600_ values must be within the dynamic range of equipment to maintain linearity in the Beer–Lambert law. However, adjustment in dilution would increase the complexity of operation and prevent quick evaluation for cell growth in the actual measurements. The dilution factor of 10 was used as the fixed value to estimate cell density, especially for the late stage of cultivation. As described above, the OD_600_ values at the end of cultivation (30 h) were out of the dynamic range in the cases of haploid and diploid strains (Fig. 1A and S2).

It was found that the OD_600_ values at growth saturation obviously decreased as the apparent cell diameter increased despite underestimation of the OD_600_ values of haploid and diploid strains. In the calculation of cell density *c*, overestimation of the proportionality constant *k* and underestimation of the OD_600_ values can partially cancel out each other. The* c* values also decreased as the apparent cell diameter (Fig. S3).

Depending on the size and shape of microbial cells, various approximations have been used to estimate the scattering intensity^1,19–21^. It depends on the radius of the scatterer, wavelength of incident light and refractive indices of the scatterer and the media, respectively^1^. Whereas the proportionality constants *k* significantly depended on the bacterial cell size^22^, there was relatively less difference in the constants *k* between yeast strains in the current study.

Unfortunately, within dynamic range in the OD_600_ measurements, I did not confirm statistically significant difference in the proportionality constants *k* between yeast strains (with different diameters). As described above, each cell suspension had heterogeneity in diameter, and the apparent cell diameter of each strain was determined to understand the characteristics of each strain. Similarly, the proportionality constants *k* at growth saturation were used to investigate the relationship with the apparent cell diameters. Importantly, it was confirmed that the constant* k* in Eq. 2 is inversely proportional to the apparent cell diameter *d* although the relationship is just an approximation. It might be rough estimation but would provide reference information for comparison in cell density between yeast strains with different diameters.

In conclusion, it was found that the apparent cell diameter is one of most important factors to estimate yeast cell density in the OD_600_ measurements based on the Beer–Lambert law. Even in the case where cell aggregation occurred in culture, the OD_600_ values were approximately proportional to the product of the apparent cell diameter and density. The approximation formula constructed in the current study would serve as a major reference source for researchers and technical experts.

## Methods

### Strains and media

Detailed information regarding *S. cerevisiae* laboratory yeast strains is shown in Table 1. Yeast cells were grown in YPD medium (1% yeast extract, 2% peptone, and 2% glucose). A final concentration of 2% agar was added to the liquid media when preparing solid media.

### Determination of cell growth characteristics

Each yeast strain was grown in 2 ml of YPD medium at 30 °C, setting initial OD_600_ at 0.1. The OD_600_ values of 10 times diluted cultures were monitored using a photoelectric colorimeter (Biowave CO8000; Biochrom Ltd., Cambridge, UK). To investigate cell density, yeast cell cultures were diluted (setting OD_600_ at 10^–5^), and then spread on the YPD solid medium. After incubation at 30 °C for 2 days, colony counting was carried out according to the previous study^9^. Three independent experiments were carried out. The statistical analysis of the *F*-test and Student’s *t*-test was applied to ascertain whether there was a significant difference in the proportionality constants *k* between yeast strains with different cell size.

### Microscopic observations

Each yeast strain was cultured at 30 °C in YPD medium for 30 h. The cell cultures were then diluted with distilled water and observed using a fluorescent microscope (BZ-9000SP, Keyence Co., Ltd., Osaka, Japan). Each image was photographed with the same exposure time using a 40 × objective lens.

### Flow cytometric analysis

The FSC of yeast cells were measured using an BD FACSMelody flow cytometer (Becton, Dickinson and Co., Franklin Lakes, NJ, USA), and the data was analyzed using FlowJo software (TreeStar, Ashland, Oregon, USA). The FSC signal was collected from 1.0 × 10^4^ cells, and the mean was determined. To draw calibration curve, 3.0 and 7.5 µm beads (Becton, Dickinson and Co., Franklin Lakes, NJ, USA), and 5.1, 15.1 and 20.0 µm beads (Spherotech Inc., Lake Forest, IL, USA) were used as size reference beads.

## Supplementary Information

Supplementary Information.

## Data Availability

The datasets used and/or analysed during the current study available from the corresponding author on reasonable request.
