# Supplementary material for: Apparent diameter and cell density of yeast strains with different ploidy
Source: Sci Rep. 2023 Jan 27;13:1513. doi: 10.1038/s41598-023-28800-z (PMC9883461; doi:10.1038/s41598-023-28800-z)
Supplement: Supplementary file 1 — Supplementary Information. [file 41598_2023_28800_MOESM1_ESM.doc]

**Supporting Information**

**Apparent diameter and cell density of yeast strains with different ploidy**

Nobuo Fukuda^1^*****

^1^Biomedical Research Institute, National Institute of Advanced Industrial Science and Technology (AIST), Osaka, Japan

*Corresponding author

Fax: +81 72 751 8370; Tel: +81 29 849 1458

E-mail: nob-fukuda@aist.go.jp

**Table S1. Deference in the proportionality constant *k* of each strain between 12 h and 30 h cultivation.**

Strain Ploidy Raw value of OD_600_ at 30 h *k_30 h_* / *k_12 h_* Degree of overestimation of *k_30 h_*

BY4741L haploid 1.01 1.47 Not Determined

BY4742L haploid 0.98 1.64 1.39

BY4743L diploid 0.87 1.23 1.30

BY4743-3L triploid 0.79 1.13 Not Determined

BY4743-4L tetraploid 0.73 1.20 Not Determined


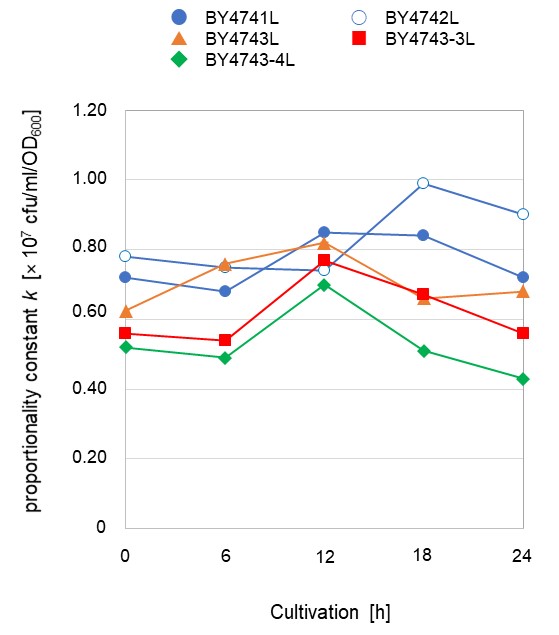


**Figure S1. Semi-quantitative evaluation for the proportionality constant *k*.** Yeast cell cultures were diluted (setting OD_600_ at 10^-3^), and 10 µl of each cell suspension was spotted on the YPD solid medium. Cell density was roughly estimated by colony counting, and proportionality constants *k* were calculated according to Equation 2.


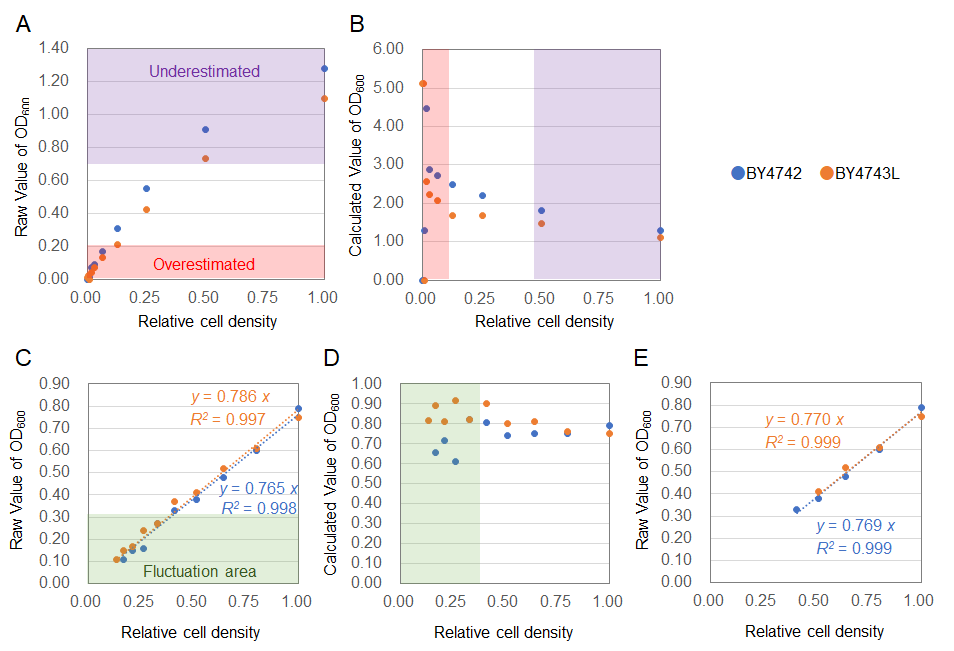


**Figure S2. Investigation of dynamic range in the OD_600_ measurements.** (A) Raw values in the OD_600_ measurement using 2-fold dilution series. (B) Calculated values of OD_600_ using the dilution factor of each cell suspension. Underestimated and overestimated OD_600_ values were shown with violet and red colours, respectively. (C) Calibration curves using 1.25-fold dilution series. (D) Calculated values of OD_600_ using the dilution factor of each cell suspension. The OD_600_ values in fluctuation were shown with green colours. (E) Calibration curves by excluding the data points in fluctuation area shown in C and D.

**
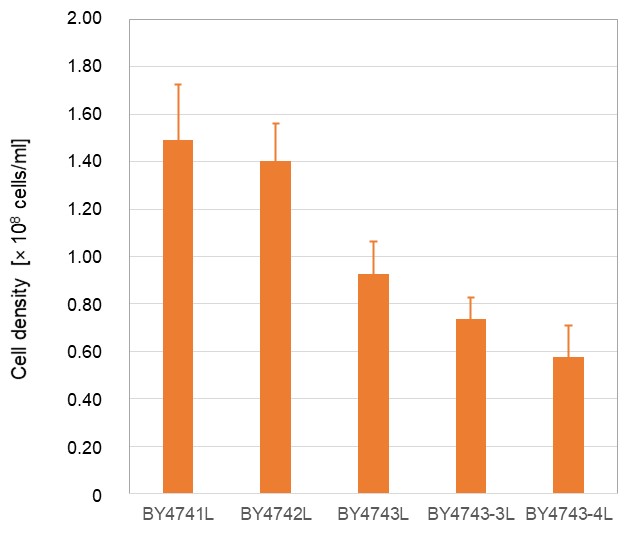
**

**Figure S3. Cell density of each strain at growth saturation.** According to Equation 2, cell density was estimated using the calculated OD_600_ and *k* values. Values are presented as means ± standard deviations from three independent experiments.

**
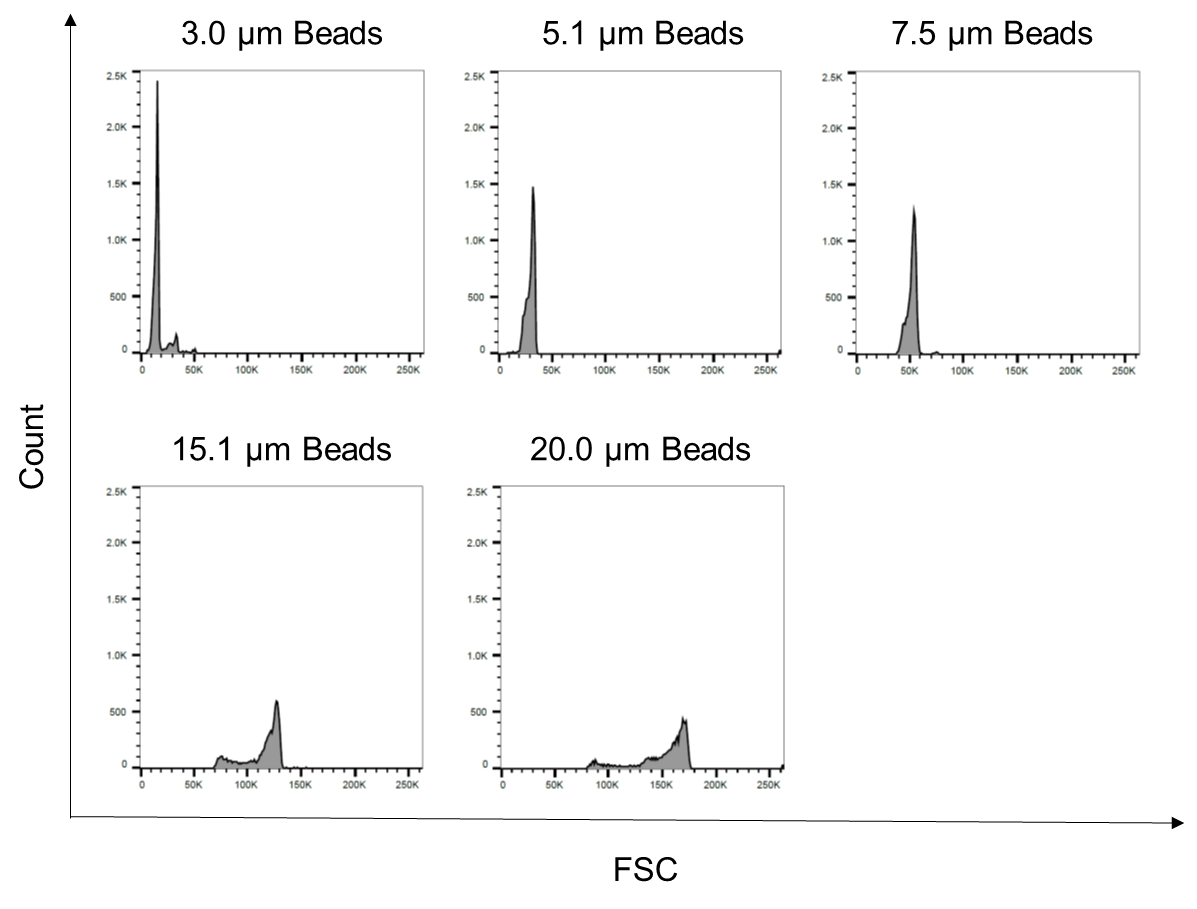
**

**Figure S4. FSC histograms of size reference beads.** Each diameter was the value provided by the reagent manufacturer.


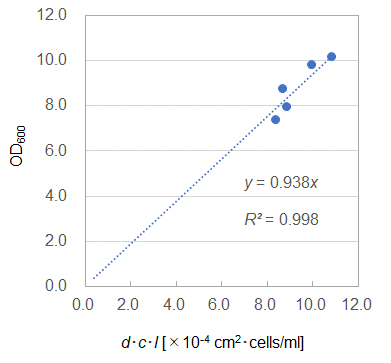


**Figure S5. Determination of the constant value in Equation 4.** Each data point represents the mean value of BY4741L, BY4742L, BY4743L, BY4743-3L and BY4743-4L, respectively.


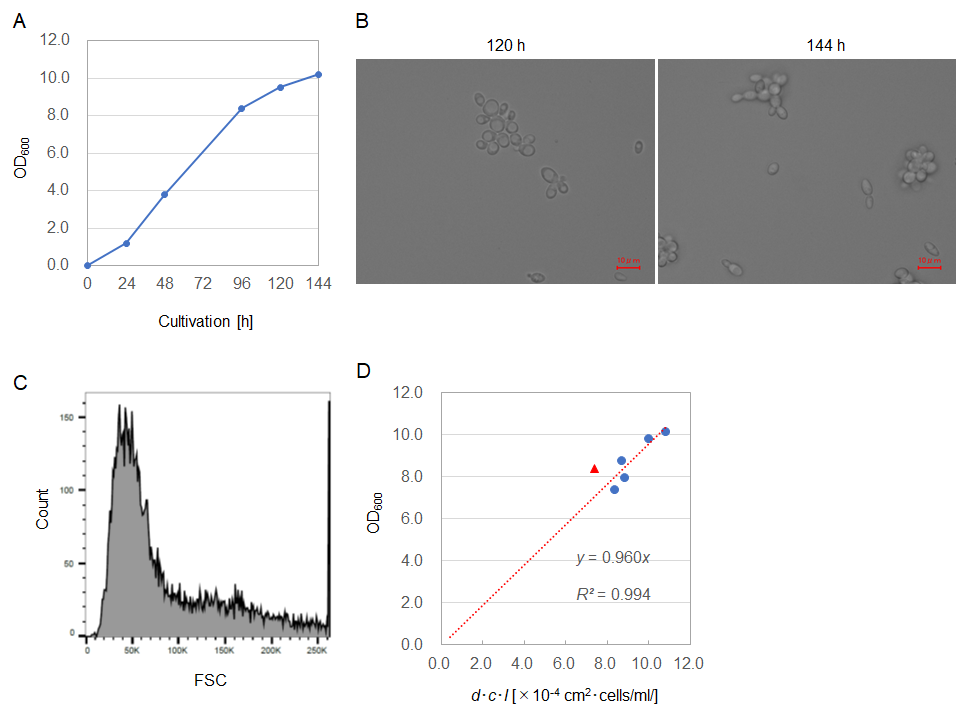


**Figure S6. Verification of Equation 4 using the sake yeast strain K7A.** (A) Growth curve. (B) Microscopy images of yeast cells. Scale bar: 10 μm. (C) FSC histogram in flow cytometric analysis (D) Investigation of the constant value in Equation 4 using the sake strain K7A (a red triangle). Blue circles represent the mean values of BY4741L, BY4742L, BY4743L, BY4743-3L and BY4743-4L, respectively.
